# The Effects of Shear Force-Based Processing of Lipoaspirates on White Adipose Tissue and the Differentiation Potential of Adipose Derived Stem Cells

**DOI:** 10.3390/cells11162543

**Published:** 2022-08-16

**Authors:** Andreas Eigenberger, Oliver Felthaus, Thomas Schratzenstaller, Silke Haerteis, Kirsten Utpatel, Lukas Prantl

**Affiliations:** 1 Department of Plastic, Hand and Reconstructive Surgery, University Hospital Regensburg, Franz-Josef-Strauss-Allee 11, 93053 Regensburg, Germany; 2 Medical Device Lab, Regensburg Center of Biomedical Engineering (RCBE), Faculty of Mechanical Engineering, Ostbayerische Technische Hochschule Regensburg, Galgenbergstraße30, 93053 Regensburg, Germany; 3 Institute for Molecular and Cellular Anatomy, University of Regensburg, Universitätsstraße 31, 93053 Regensburg, Germany; 4 Institute of Pathology, University of Regensburg, Franz-Josef-Strauss-Allee 11, 93053 Regensburg, Germany

**Keywords:** white adipose tissue, lipoaspirate, lipograft, fat grafting, stem cells, surgery, cell-enriched lipotransfer, CELT

## Abstract

Autologous lipotransfer is a promising method for tissue regeneration, because white adipose tissue contains a heterogeneous cell population, including mesenchymal stem cells, endothelial cells, immune cells, and adipocytes. In order to improve the outcome, adipose tissue can be processed before application. In this study, we investigated changes caused by mechanical processing. Lipoaspirates were processed using sedimentation, first-time centrifugation, shear-force homogenization, and second-time centrifugation. The average adipocyte size, stromal vascular cell count, and adipocyte depot size were examined histologically at every processing step. In addition, the adipose derived stem cells (ADSCs) were isolated and differentiated osteogenically and adipogenically. While homogenization causes a disruption of adipocyte depots, the shape of the remaining adipocytes is not changed. On average, these adipocytes are smaller than the depot adipocytes, they are surrounded by the ECM, and therefore mechanically more stable. The volume loss of adipocyte depots leads to a significant enrichment of stromal vascular cells such as ADSCs. However, the mechanical processing does not change the potential of the ADSCs to differentiate adipogenically or osteogenically. It thus appears that mechanically processed lipoaspirates are promising for the reparation of even mechanically stressed tissue as that found in nasolabial folds. The changes resulting from the processing correspond more to a filtration of mechanically less stable components than to a manipulation of the tissue.

## 1. Introduction

Autologous fat transfer, also known as lipofilling, is a common procedure in plastic and aesthetic medicine in order to treat volume and contour defects in soft tissues [1,2]. However, since the identification of adipose derived stem cells (ADSCs), and stromal vascular cells (SVCs), the spectrum of applications for tissue regeneration has expanded rapidly. For example, the technique is used to treat Dupuytren’s contracture, systemic sclerosis, and chronic wound healing [3]. In vitro, it has already been demonstrated that ADSCs can differentiate adipogenically, chondrogenically, osteogenically, and neurogenically [4,5,6]. This and the fact that ADSCs can be minimally invasively harvested from any subcutaneous white adipose tissue (WAT) makes ADSCs a promising tool in clinical tissue regeneration.

Unfortunately, clinical results are currently inconclusive because each research group implements different processing protocols that are not often precisely described [7,8,9]. In the literature, reabsorption rates between 20 to 90% are given [6]. Some groups only remove the red layer through centrifugation or filtration. Others try to increase the number of ADSCs in the graft through enzymatic or mechanical processing. However, if ADSCs are to be injected intravenously, for example as therapy in neurodegenerative disorders, the adipose tissue must be completely digested [10]. Which protocol is ultimately used depends not only on the therapeutic aim, but also on the time required for processing and the varied legal regulations worldwide.

Although high stem-cell yields can be achieved by digesting adipose tissue [11], there may also be advantages to leaving ADSCs in their native microenvironment after mechanical processing. Additionally, non-enzymatic processing is considered more efficient and safer, as digestion may induce local inflammatory reactions owing to the activation of human complements [12,13]. What happens to the lipoaspirate during mechanical processing remains largely unexplored.

Prantl et al. have already demonstrated that shear force processing does not change the secretome of ADSCs [14]. In our study, we investigate what changes in the composition of white adipose tissue following centrifugation and from the mechanical processing force. Having focused exclusively on ADSCs to date, the effects of mechanical processing have not yet been studied for the adipocytes and the entire WAT. We hypothesize that mechanical processing through shear forces dissolves the large adipocyte depots, but mechanically stable structures such as connective tissue, SVCs, and adipocytes with pericellular fibrotic structures remain unchanged. Neither centrifugation nor homogenization should affect the differentiation ability of ADSCs.

## 2. Materials and Methods

### 2.1. Study Design and Patient Demographics

Lipoaspirates were obtained following the informed consent from 11 healthy women aged on average 39 years (range from 25 to 62), who underwent liposuction for aesthetic reasons. The BMI of the patients averaged 29.6 ± 5.2 kg/m². The most frequent region of aspiration was the lower extremities (*n* = 7), followed by the abdomen (*n* = 2) and upper extremities (*n* = 2). The ethics review board of the University Hospital in Regensburg approved the study (08/117).

### 2.2. Liposuction Technique

In preparation for liposuction, a physiological saline solution (0.9% *w*/*v*) containing adrenaline at a concentration of 1:100,000 was infiltrated using the body-jet evo and a 2.5 mm injection cannula (both Human Med AG, Schwerin, Germany). According to the S2K guidelines, 15 min of exposure time passed before liposuction was performed [15]. Subsequently, the harvesting cannulas with a diameter between 3.5 and 4.2 mm (Human Med AG) were connected to the body-jet, which ensured an even negative pressure of less than 0.5 mbar.

### 2.3. Sample Preparation

**Figure 1:**
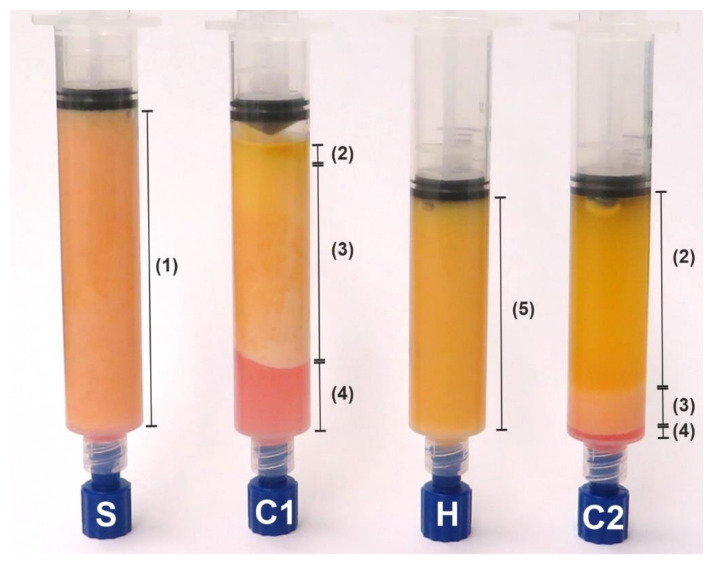
Macroscopic appearance of the lipoaspirate after each processing step reveals the volume decrease and lightening of the fat phase. Starting on the left: sedimented (S), first centrifugation (C1), homogenized (H), and second centrifugation (C2). Following steps S and H, the homogeneous phases appear (1 and 5), whereas after steps C1 and C2, an oily phase (2), a fat phase (3), and a red layer (4) can be distinguished. It should be noted that after steps C1 and C2, the red layer and oily phases, respectively, were discarded and only the fat phase was processed further.

Once harvesting was completed, the collected lipoaspirates were inserted into 20 mL syringes (Becton Dickinson GmbH, Heidelberg, Germany) using a ø3.0 mm cannula (Aesthetic Group, Puiseux-le-Hauberger, France). The syringes were then centrifuged for 2 min at 1600 rcf using the Rotina 380R (Andreas Hettich GmbH & Co.KG, Tuttlingen, Germany). Subsequently, the discontinued red layer was discarded. The fat phase was then homogenized, in which the fat tissue was forced manually through a multidirectional stopcock (B. Braun Melsungen AG, Melsungen, Germany) ten times using two 10 mL syringes (Becton Dickinson GmbH). In order to separate the released lipids from the remaining fat tissue, we again centrifuged the lipoaspirates at 1600 rcf for 2 min. For further analysis, 3 mL aliquots of the fat tissue were taken before (S) and after the first centrifugation (C1), after homogenization (H), and after the second centrifugation (C2). Figure 1 illustrates the appearance of the lipoaspirate after each of the processing steps.

### 2.4. Fixation and Staining

All tissue samples were fixed in a neutral buffered formalin and embedded in paraffin. Histological slides with a thickness of 4 µm were prepared, deparaffinised with ethanol and xylene, and then stained according to the standard protocol for hematoxylin and eosin. Subsequently, all samples were scanned using a P1000 (3D Histech Kft., Budapest, Hungary).

### 2.5. Histological Sample Evaluation

The samples were evaluated with CaseViewer (3DHISTECH Kft., Budapest, Hungary). In order to quantify the differences between the processing steps, we recorded the largest diameter of the adipocytes, the number of adipocytes directly adjacent to each other (adipocyte depots), and the number of stromal vascular cells (SVCs). In order to ensure standardization, all sample measurements were performed according to the same parameters, which Table 1 summarizes:

In order to ensure a high degree of heterogeneity, the diameter of the ten adipocytes at the top of each image per area was measured. Since there were not always ten adipocytes per area, the number of examined areas varied between the samples. For all measurements, the areas were picked randomly using the mini map. If the area did not contain any cells or extracellular matrix (ECM) at all, we excluded that area from the analysis. The area size was measured using ImageJ (National Institutes of Health, Bethesda, MD, USA).

In addition to the quantitative evaluation, a qualitative description of the samples is also provided in the results section.

### 2.6. ADSC Isolation and Differentiation

For three randomly chosen patients, the adipose tissue was enzymatically digested after each of the four processing steps, as described previously [14,16]. In summary, 1 mL of α-minimal essential medium (Gibco Thermo Scientific, Waltham, MA, USA) containing 0.2% (*w*/*v*) collagenase (from Clostridium histolyticum; Sigma Aldrich, St. Louis, MO, USA) was added to 0.75 mL of the processed lipoaspirates. Following the incubation for 45 min at 37 °C, the samples were centrifuged at 500 rcf for 5 min. Subsequently, the supernatant was discarded, the cells were resuspended, and seeded into T25 cell culture flasks. There, the cells grew for three days at 37 °C and 5% carbon dioxide in a humidified atmosphere. Once the cells had grown to confluence, they were detached from the culture flask using trypsin (Sigma Aldrich) and counted using a Neubauer counting chamber. Samples were seeded in quadruplicates with 3750 cells per well. A separate 48-well plate was used for each differentiation.

Once the cells were 80–90% confluent, a supplemented medium for adipogenic differentiation was added to one plate and a supplemented medium for osteogenic differentiation was added to the other. The ingredients for both media are listed in Table S1 (Supplementary Materials). Following the regular medium changes for 14 days, the adipogenic differentiation was stopped. The cells were fixed with formalin (Merck KGaA, Darmstadt, Germany) and stained with an oil red solution (Sigma Aldrich) in order to reveal the adipocytes. The osteogenic differentiation was stopped after five weeks. Subsequently, the cells were fixed using formalin (Merck KgaA) and stained using alizarin red S (Sigma Aldrich). The plates were carefully washed prior to quantification. The optical density was measured at 510 nm for the adipogenic differentiation and at 540 nm for the osteogenic differentiation using a VarioScan (Thermo Scientific, Waltham, Waltham, MA, USA).

### 2.7. Statistical Analysis

We initially carried out an analysis of variance (ANOVA). If the results proved to be significant (*p* < 0.05), we used Tukey’s honestly significant difference post hoc test (if applicable). For better readability, the confidence intervals are not given. All calculations and graph plots were generated using R 4.1.0 (R Core Team, Vienna, Austria) (4.1.0) and R Studio (RStudio Inc., Boston, MA, USA).

## 3. Results

### 3.1. Quantitative Description of the Histological Samples

**Figure 2:**
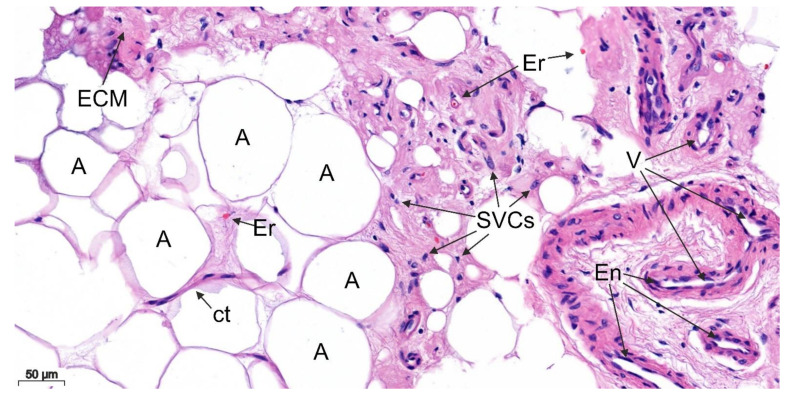
Overview of the individual components of the harvested WAT, containing different sized adipocytes (A), areas full of the extracellular matrix (ECM), connective tissue (ct), erythrocytes (Er), vessels (V), endothelial cells (En), and other SVCs.

We were able to analyze a total of 44 samples, following the successful preparation, fixation, and staining of the samples. Regardless of the processing step, the adipocytes, larger accumulations of the ECM, the erythrocytes, the vessels with their endothelial cells, and other SVCs could be detected in all samples. An example of this is illustrated in the following figure (Figure 2). The SVCs are a heterogeneous cell population containing ADSCs, pre-adipocytes, endothelial cells, pericytes, and immune cells such as macrophages or T cells [17].

**Figure 3:**
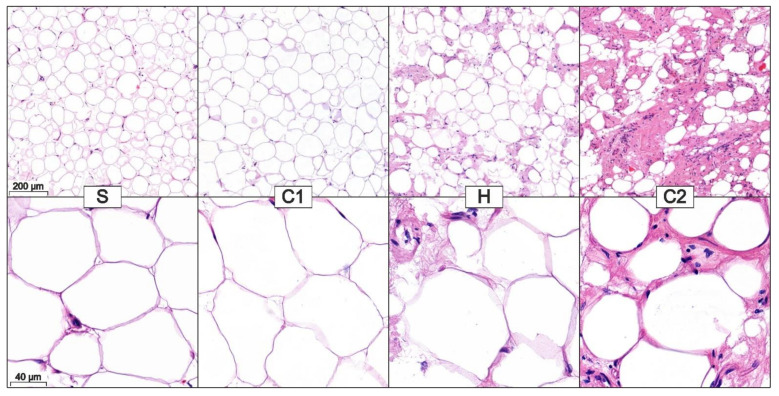
Microscopic appearance of the lipoaspirate after sedimentation (S), first centrifugation (C1), homogenization (H), and second centrifugation (C2) reveals a decrease in the adipocyte depots and an increase in the ECM surrounding the single adipocytes. The shape of the adipocytes does not change during processing.

Nevertheless, even at a low magnification, the differences between the processing steps can be noticed: Samples S and C1 comprise large depots of adipocytes containing little ECM (Figure 3) and large accumulations of the ECM containing few adipocytes (Figure S1, Supplementary Materials). However, in the samples H and C2, small adipocyte accumulations surrounded by a lot of ECM can be seen. In these samples, no pure ECM or adipocyte depots can be identified.

At a higher magnification (20×), the adipocytes in the different samples have a comparable shape. Occasionally, darker staining can be seen between the lighter stained adipocyte membranes. In some places, the nucleus of the adipocytes was cut. Since we examined WAT, all samples contained only univacuolar adipocytes.

It should be noted that both S and C1 and H and C2, respectively, are most similar at both magnification levels.

### 3.2. Stromal Vascular Cells

**Figure 4:**
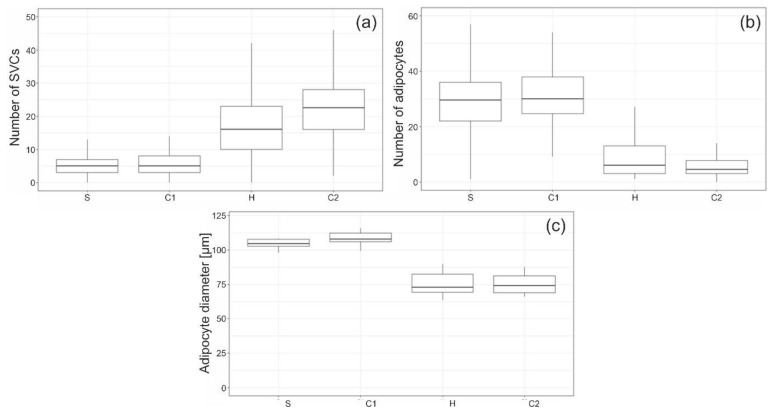
Quantitative comparison of all four processing steps regarding the counted number of SVCs in an area of 0.03 mm² (**a**), the number of adipocytes in an area of 0.31 mm² (**b**), and the mean adipocyte diameter (**c**).

Following processing (C2), the number of SVCs per defined area was 3.7 times higher than before processing (S). The values for S and C1 on the one hand, and H and C2 on the other hand are similar. The differences between the pre- and post-homogenization steps portrayed in Figure 4a are significant (*p*-values < 0.001). The individual components of SVCs could not be identified adequately basing solely on the histological images.

### 3.3. Adipocyte Depots

In order to quantify the size of adipocyte accumulations, we counted the number of adipocytes directly adjacent to each other in an area of 0.31 mm² (Figure 4b). For each processing step, 110 areas sized 0.31 mm² were counted. In all processing steps, there were areas with a small number of contiguous adipocytes. However, the difference before (S and C1) and after homogenization (H and C2) proved to be significant (*p*-values < 0.001).

### 3.4. Adipocyte Size

In each processing step, 1100 adipocytes were counted, which were evenly distributed in all patients. The mean diameter of the largest adipocyte in samples S and C1 (pre homogenization) was significantly smaller than in H and C2 (post homogenization). The *p*-value was < 0.001 for all pairings except S and C1 and H and C2. The distribution is portrayed in Figure 4c.

For each patient, the mean adipocyte size decreased significantly about 28.3 ± 7.7% between S and H and 28.7 ± 5.3% between S and C2 (*p*-values both < 0.001). The difference between S and C1, and between H and C2 was not significant (*p*-values = 0.731 and 0.999).

**Figure 5:**
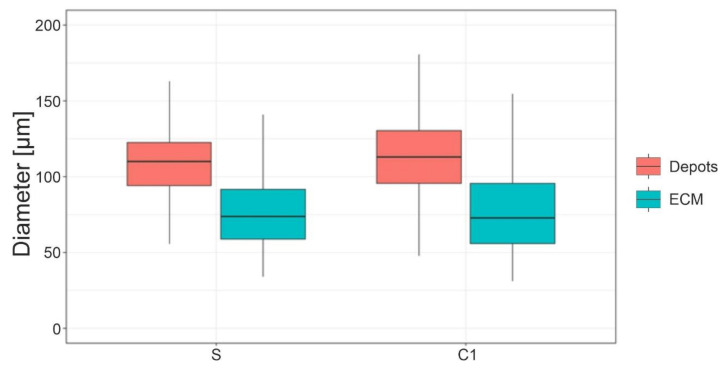
Comparison of adipocyte diameters according to whether they are located in adipocyte depots or near the ECM accumulations in the processing steps S and C1.

In S and C1, the adipocytes exclusively adjacent to other adipocytes and those that border on the ECM accumulations can be distinguished. As Figure 5 below depicts, the diameters of adipocytes at stages S and C1 that border on the ECM accumulations are significantly smaller than those of adipocytes in depots (*p*-values < 0.001). It should be noted that the diameter of these smaller adipocytes is similar to the diameter of adipocytes found in H and C2, where no adipocyte depots were found. For each processing step, 440 adipocytes were measured.

### 3.5. Adipogenic Differentiation

**Figure 6:**
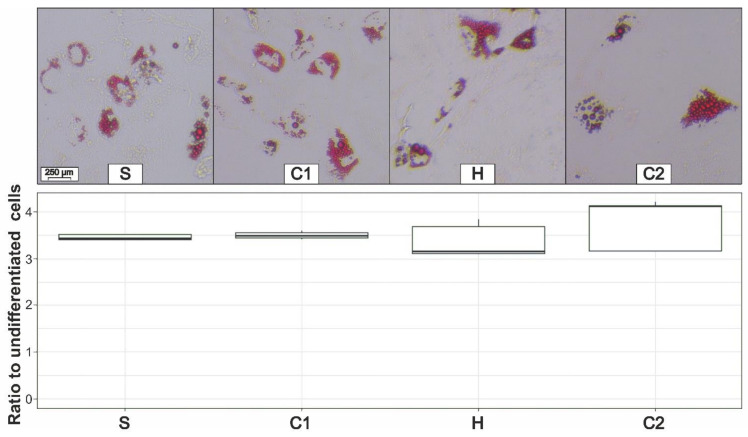
Microscopic comparison of the adipogenic differentiation after the oil red staining (**top**) and the quantification of the adipogenic differentiation of ADSCs after sedimentation (S), first centrifugation (C1), homogenization (H), and second centrifugation (C2) (**bottom**) in ratio to the optical density of the undifferentiated ADSCs.

Adipogenic differentiation was first examined quantitatively using a microscope. The adipogenic differentiation of ADSCs was successful in all four preparation phases. No qualitative differences between the individual phases could be detected (Figure 6). In order to improve the comparability of the quantification, the seeded cell number was equal for all four processing steps. We also analyzed the ratio between the differentiated cells and the average optical density of undifferentiated cells (control group). The differences between the individual groups are portrayed in Figure 6 and are not significant (*p*-value = 0.39).

### 3.6. Osteogenic Differentiation

**Figure 7:**
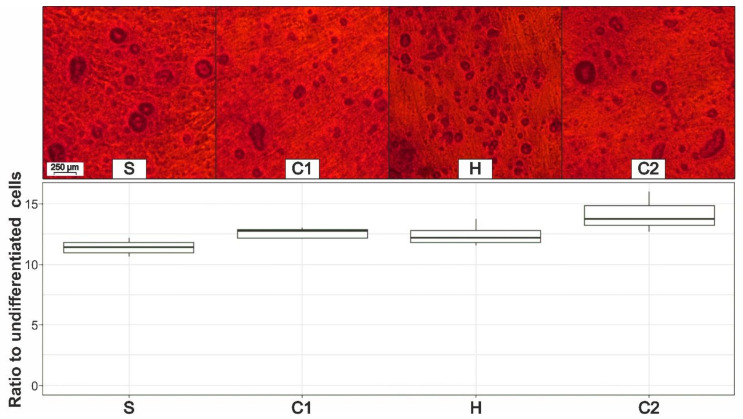
Microscopic comparison of the calcific deposits after the osteogenic differentiation and the alizarin red S staining (**top**) and the quantification of the osteogenic differentiation of the ADSCs after sedimentation (S), first centrifugation (C1), homogenization (H), and second centrifugation (C2) (**bottom**) in ratio to the optical density of the undifferentiated ADSCs.

Osteogenic differentiation was also successful in all samples, which is revealed by the alizarin red S staining of calcific deposits in Figure 7. No microscopic differences between the individual preparation steps could be identified. In order to improve the comparability of the quantification, the seeded cell number was equal for all four processing steps. Again, we analyzed the ratio between the differentiated cells and the average optical density of undifferentiated cells (control group). The differences between the individual groups are portrayed in Figure 7 and are also not significant (*p*-value = 0.12).

## 4. Discussion

In all preparation steps, we were able to detect adipocytes, larger accumulations of the ECM, erythrocytes, vessels with their endothelial cells, and other SVCs. However, there are significant differences between the processing steps. These become apparent just by viewing the histological scans but they were confirmed in each case through quantitative evaluation.

The decrease in adipocyte size (Figure 4c) is caused by homogenization but not centrifugation. However, this decrease in adipocyte size is not due to a change in the remaining adipocytes since their shape did not change during centrifugation or homogenization. Additionally, all measured adipocyte diameters are within the range of values already reported in the literature [18,19]. The fact that the mean value decreases is because the larger adipocytes in the depots are removed by homogenization, while adipocytes in the proximity of the ECM remain unaffected (see Figure S2, Supplementary Materials). Sabarti et al. found that different types of white adipose tissue can be differentiated based on structural features [20]. Furthermore, Comley et al. discovered that WATs differ in their toughness and in their resistance to mechanical forces. It has been suggested that these properties are determined by the adipocyte size and the surrounding collagen network [21]. This hypothesis can be confirmed by our experiments. While the adipocytes in depots do not withstand the shear forces of homogenization, the adipocytes surrounded by the ECM remain unchanged. In native adipose tissue, fat depots occur primarily in tissues that are not mechanically stressed, whereas the ECM-rich, fibrous white adipose tissue (fWAT) occurs in areas that are exposed to increased mechanical stress [20].

The size of the ECM near adipocytes at S and C1 (Figure 5) is similar to the mean adipocyte size of H and C2 (Figure 4c). The significantly larger adipocyte diameter at S and C1 in Figure 4c is due to the adipocytes being larger in the depots than near the ECM, which is congruent with the findings of Kruglikov et al. [22]. The disruption of adipocyte depots by shear forces could also be demonstrated quantitatively by counting the adipocytes lying next to each other. This number decreases considerably following homogenization but not centrifugation. We must note that the number of adipocyte depots should be larger than counted, since the area of 0.31 mm² was the limiting factor here. The disrupted adipocytes release their stored lipids, which is separated from the fat phase by a second centrifugation [23,24]. The red layer and oily phases contain far fewer vital cells and growth factors [25]. Therefore, they are discarded during the processing and just the pure fat phase is used for transplantation.

When regenerating tissue through an autologous fat transfer, it is advisable that the tissue used has the composition and mechanical properties comparable to the target tissue [20]. We have demonstrated in our study that, through a simple surgical protocol, the mechanically more stable WAT can be isolated through mechanical forces alone. The fact that the remaining adipocytes are not altered, but adipocytes in proximity to the ECM are present in all phases, is shown in Figure S2 (Supplementary Materials). This gives us the opportunity to combine two advantages. First, for example, the easily suctioned and depot-rich subcutaneous WAT can be used from the abdomen or thigh. Second, we can transplant the mechanically stable fat (after the second centrifugation) with properties comparable to those of the target tissue, thereby leading to satisfying long-term results. This has already been demonstrated in one of our clinical studies on facial lipofilling, offering good long-term results over one year, even in areas of mechanical stress such as the nasolabial fold or periorbital area [26].

In addition to the improved mechanical properties, the completely processed lipoaspirates (after C2) has two other advantages. The increased number of SVCs and thus endothelial cells leads to the improved neoangiogenesis of the implanted tissue [27,28,29] and the smaller particle size after homogenization reduces the diffusion distance, resulting in better take rates and less necrosis [30]. It should be noted that the increase in SVCs (Figure 4a) results from the volume loss of adipocyte depots (Figure 1 and Figure 3) and not from cell proliferation [14].

Graft survival depends on both neovascularization and in vivo cell proliferation. Therefore, the number of ADSCs in the graft is particularly important, as both neovascularization and proliferation are facilitated by growth factors secreted by the ADSCs [14,31,32]. The increase in the SVCs by a factor of 3.7 is comparable to the increase in the ADSCs from the processing [14,26,33]. This has already been shown in several studies that used comparable processing protocols.

Our study demonstrates that the ability of mechanically processed ADSCs to differentiate adipogenically or osteogenically was not altered by centrifugation or homogenization. This was demonstrated both qualitatively and quantitatively. Interestingly, in a recently published study, Deng et al. demonstrated that the mechanosensing of ADSCs is interconnected via intracellular Ca^2+^. Increased Ca^2+^ levels lead to an activation of osteogenically related genes [34]. Whereas the mechanical stress during the experiments of Deng et al. was constant, it was limited in our experiments due to the short duration of centrifugation and homogenization. It seems that the short-term stress does not lead to the sustained activation of osteogenic genes, as we did not observe any differences between the mechanically unstressed sample S and samples C1, H, and C2. In vitro, ADSCs can also differentiate into vascular smooth muscle cells, chondrogenic cells, and neuron/motor neuron-like cells [35,36,37,38]. Nevertheless, we focused on the two established laboratory protocols for ADSC differentiation. Prantl et al. revealed that the shear forces do not lead to a change in the secretion ability of ADSCs [14]. Summarizing this, the fact that the shape of the adipocytes is not changed through the processing, just as the differentiation potential of the ADSCs is not altered by centrifugation or homogenization, allows us to conclude that the mechanical processing constitutes no substantial manipulation of the lipoaspirates. The shear forces only cause the mechanically unstable adipocyte depots to break down and be removed by centrifugation.

Prantl et al. were able to show in patients with benign lipomatosis that the affected adipose tissue has more SVCs and ADSCs, smaller adipocytes, and a higher cellular proliferation rate [39]. However, our standardized differentiation experiments revealed that the differentiation potential and proliferation rate did not change after the mechanical processing. Even though the differentiation potential for each individual ADSC remains unchanged, it should be noted that there are many more cells capable of differentiation in mechanically processed tissue.

In addition to the mechanical processing of adipose tissue, enzymatic processing has also become established. One advantage of enzymatic processing is that high numbers of ADSCs can be obtained [11,12,40]. However, such ADSCs are detached from their cell association and the adipocytes, and the ECM is discarded. The mechanically processed ADSCs are less concentrated but remain in their niches, which may be the stimulus required in order to proliferate and regenerate in the target tissue [41]. Furthermore, the extracellular matrix and its components may also play an important role in regenerative treatment [33]. In comparison, mechanical processing is faster, cheaper, and less risky [12,42], which makes it the most promising way to repair even mechanically stressed tissue with fat stem cells.

A limitation of our study is that the only participants were female subjects. Owing to the fact that many more female patients undergo liposuction, several other research groups have also limited their focus to female subjects [43,44,45]. Another limitation of this study is the lack of any animal experiments, for example, to investigate the osteogenic differentiation capability of the cells for bone repair after the different processing steps. Final conclusions about the ability of the processed lipoaspirates for tissue regeneration can only be drawn after animal experiments. However, the effect of the processed lipoaspirates in homologous use has already been clinically demonstrated and indicates tissue regeneration [26].

Since the mechanically prepared graft has been shown to have a number of advantages over the pure isolated ADSCs, it would be very interesting to compare the effects on bone healing using these two methods in the future. Additionally, in further experiments, we will investigate the impact of different types of stress on the aging of isolated ADSCs. To this end, quantitative analyses of high β-galactosidase levels should be performed. Now that the secretory properties of adipocytes are increasingly becoming the focus of studies [46], they should also be investigated soon with respect to the WAT classification and the effects of mechanical processing.

## 5. Conclusions

Lipoaspirates can be mechanically processed in order to enrich the yield of stromal vascular cells and mesenchymal stem cells. However, mechanical processing does not affect the potential of the stem cells to differentiate or change the shape of remaining adipocytes. Since the processing discards mechanically unstable portions of the tissue, the processed lipoaspirates may be used particularly well in mechanically stressed target regions. This should be investigated in further clinical studies.

## Figures and Tables

**Table 1:** Microscope parameters in the quantitative examination.

Measurement	Magnification	Area Size	Number of Areas per Sample
Adipocyte depots	20×	0.31 mm²	10
Adipocyte diameter	40×	0.08 mm²	>10
Number of SVCs	63×	0.03 mm²	20

## Data Availability

Not applicable.

## Supplementary Materials

The following supporting information can be downloaded at: https://www.mdpi.com/article/10.3390/cells11162543/s1, Table S1: Adipogenic and osteogenic differentiation protocol; Figure S1: Sedimented lipoaspirate portraying large adipocyte depots with less ECM and large ECM accumulations with fewer adipocytes; Figure S2: Adipocytes in proximity to the ECM in all four processing steps all demonstrate a strongly expressed cell membrane.
