# Supplementary material for: The Effects of Shear Force-Based Processing of Lipoaspirates on White Adipose Tissue and the Differentiation Potential of Adipose Derived Stem Cells
Source: Cells. 2022 Aug 16;11(16):2543. doi: 10.3390/cells11162543 (PMC9406387; doi:10.3390/cells11162543)
Supplement: Supplementary file 1 [file cells-11-02543-s001.zip › cells-1857797-supplementary.pdf]

Supplementary Matrials

Table S1. Adipogenic and osteogenic differentiation protocol:

Adipogenic differentiation			
Name	Manufacturer	End concentration	Volume/mL*
IBMX	SERVA Electrophoresis GmbH, Germany	0.5 mM	1
Dexamethasone	Sigma Aldrich, Missouri	1 µM	1
Insulin	Sigma Aldrich, Missouri	10 µM	5.7
Indomethacin	Sigma Aldrich, Missouri	200 µM	2

Osteogenic differentiation			
Name	Manufacturer	End concentration	Volume/ml
L-Ascorbic acid 2-phosphate	Sigma Aldrich, Missouri	50 µM	1 µL
Dexamethasone	Sigma Aldrich, Missouri	0.1 µM	0.1 µL
B-glycero-phosphate	Sigma Aldrich, Missouri	10 mM	5 µL

* Volume per milliliter α-Mem medium

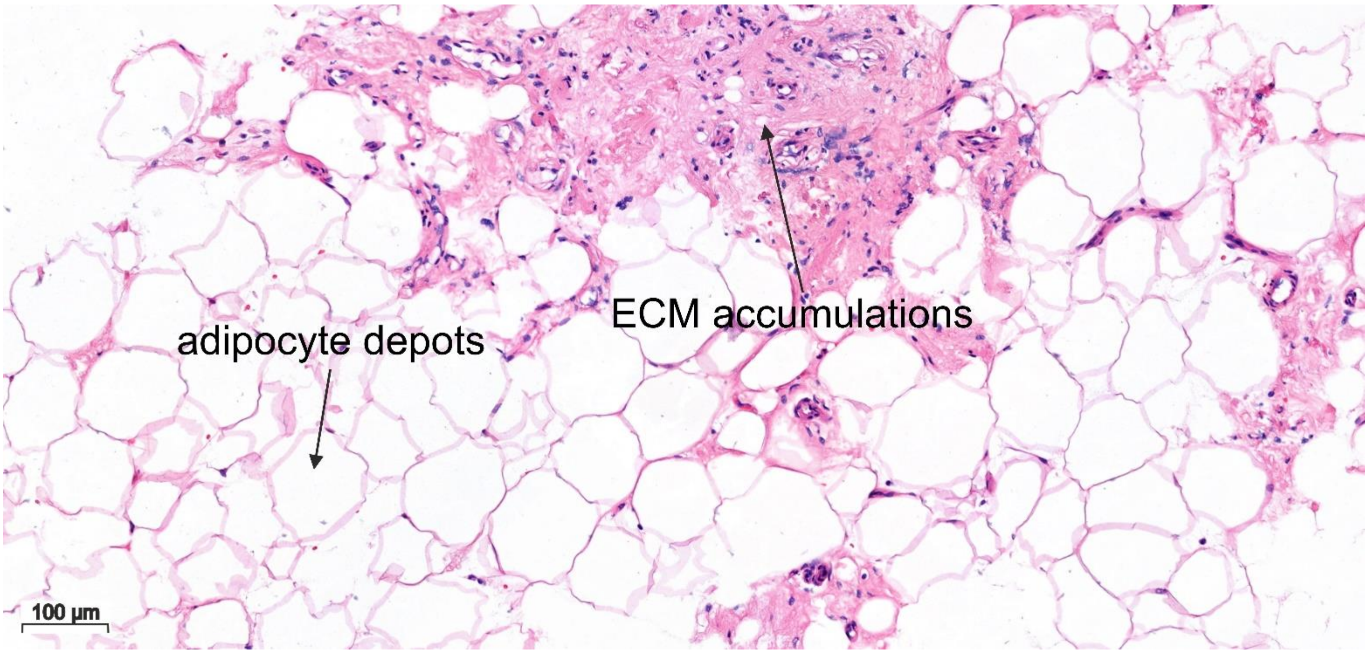

Figure S1. Sedimented lipoaspirate, portraying large adipocyte depots with less ECM and large ECM accumulations with fewer adipocytes

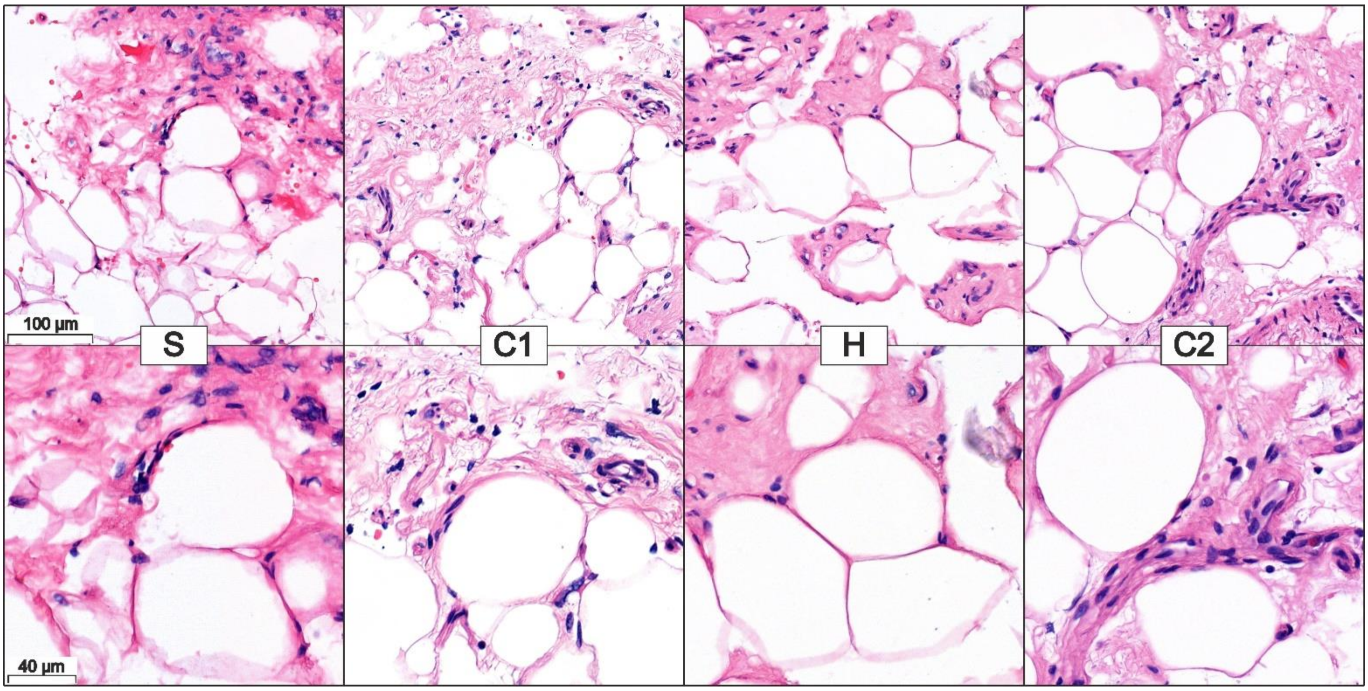

Figure S2: Adipocytes in proximity to ECM in all four processing steps all demonstrate a strongly expressed cell membrane.
